# Less Restrictive Medicaid Policies for Direct‐Acting Antiviral Access Are Associated With Greater Declines in Hepatocellular Carcinoma Deaths

**DOI:** 10.1002/cam4.71624

**Published:** 2026-02-15

**Authors:** Gabriel V. Lupu, Bhagyashree Behera, A. Sidney Barritt, Sasha Deutsch‐Link, Jane Giang, Ellen W. Green, Oren K. Fix, Neil D. Shah, Hersh Shroff, Andrew M. Moon

**Affiliations:** ^1^ Division of Gastroenterology and Hepatology The University of North Carolina Chapel hill North Carolina USA; ^2^ Department of Pharmacy The University of North Carolina Health Chapel hill North Carolina USA; ^3^ Lineberger Cancer Center The University of North Carolina Chapel hill North Carolina USA

**Keywords:** epidemiology, hepatitis C, hepatocellular carcinoma, liver, medicaid, outcomes research

## Abstract

**Background:**

In the United States (US), chronic hepatitis C virus (HCV) is the leading cause of hepatocellular carcinoma (HCC). Direct‐acting antivirals (DAAs) cure HCV and reduce HCC risk, but Medicaid DAA coverage varies across states.

**Aim:**

We assessed whether Medicaid DAA access was associated with trends in HCC‐related deaths.

**Methods:**

We analyzed CDC WONDER death certificate data (1999–2023) to assess HCC‐related mortality. US states were grouped based on Medicaid DAA prior authorization restrictions using the Hepatitis C: State of Medicaid Access scoring system: A+/A (*n* = 28), B (*n* = 11), and C/D (*n* = 12). We used NCI Joinpoint software to calculate the annual percentage change (APC) and average annual percent change (AAPC) in age‐adjusted death rate. State‐specific HCC crude death rates were analyzed before and after 2014, alongside changes in Medicaid DAA policies from 2014 to 2024.

**Results:**

Before 2017, HCC‐related death rates were positive in group A + /A (APC 2.01, 1999–2017), group B (APC 3.40, 1999–2009), and group C/D (APC 2.04, 1999–2023). Age‐adjusted death rates became negative in group A+/A states (APC −0.19, 2017–2023), while death rates continued to be positive for group B states (APC 1.49, 2009–2023) and group C/D states (APC 2.04, 2017–2023). The AAPC (1999–2023) was lowest in group A + /A (1.46), followed by group B (2.28) and C/D (2.04). From 2014 to 2024 accessibility to DAAs improved.

**Conclusion:**

Increased DAA access was associated with reduced HCC‐related death rates. Improved HCV treatment could contribute to decreased HCC incidence and recurrence, enhance linkage to subspecialty care, and prevent liver‐related decompensation.

## Graphical abstract

US states with less Medicaid restrictions for direct‐acting antivirals (DAAs) were associated with a greater decline in hepatocellular carcinoma (HCC) deaths. Policy to promote access to hepatitis C virus (HCV) treatment can improve death rates from HCC.

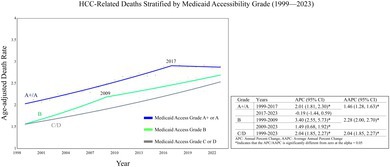

## 1 Introduction

Direct‐acting antivirals (DAAs), which result in the cure of more than 95% of patients infected with hepatitis C virus (HCV), were introduced to the market in 2014 [1]. Given the availability of highly effective, well‐tolerated treatments, the Centers for Disease Control and Prevention (CDC) recommended universal screening for HCV in all adults in 2020 [2]. Despite these recommendations, 2.4 million Americans remain infected with HCV [1]. Untreated HCV infection leads to well‐known downstream complications such as the development of chronic liver disease, cirrhosis, and hepatocellular carcinoma (HCC) [3]. The World Health Organization (WHO) had proposed total HCV elimination by 2030, but the United States (US) is not projected to meet this goal. The US Hepatitis C Elimination Plan aims to eradicate HCV in the US through improved awareness, screening, and access to treatments [1, 4]. An estimated 60% of patients infected with HCV have public insurance (Medicare, Medicaid, or other government health plan), and 44% of those are below the poverty line [5]. In the US, injection drug use is the main route for HCV transmission [1]. Given strong evidence for HCV cure in people who inject drugs (PWID), the American Association for the Study of Liver Diseases (AASLD) strongly asserts that drug use or concern for reinfection is not a contraindication for HCV treatment [6]. Access to DAA medications for Medicaid‐insured patients is subject to policy restrictions, which vary significantly from state to state [7]. Given the clear association between HCV treatment and reduction in HCC incidence and mortality, Medicaid policies on DAA access may have a direct impact on downstream statewide HCC mortality [8].

In the US, hepatocellular carcinoma (HCC) is the most common subtype of primary liver cancer and is most frequently caused by HCV‐related cirrhosis [3]. Among all cancer patients in the US, those with HCC have among the lowest 5‐year survival rates (22%) [9]. Left untreated, HCV infection leads to liver fibrosis progression, cirrhosis, and increased risk of HCC [3, 6]. HCV treatment can prevent HCC or liver‐related mortality in multiple ways: (1) halting liver fibrosis progression and incidence of HCC, (2) reducing recurrence of HCC in patients with a history of liver cancer, and (3) reducing the risk of liver‐related decompensation in patients with established HCC [10, 11, 12, 13]. With the advent of DAAs, HCV eradication has been shown to decrease the risk of developing HCC or HCC‐related death [8, 10]. Compelling evidence shows that efforts to eradicate HCV are related to this decline in HCV‐related HCC in Veteran Health Administration (VHA) data and that treatment with DAAs leads to improved overall survival [14, 15]. Patients with both chronic HCV and HCV‐related HCC have been shown to have improved liver and non‐liver outcomes when achieving SVR after DAA treatment, including mortality and HCC incidence [10, 12, 13]. SVR rates have also been shown to be above 90% in at‐risk populations such as PWID and patients with harmful alcohol use [16].

Access and affordability to DAA medications remain a major barrier to HCV eradication [7]. While there have been significant shifts in chronic liver disease etiology in the US with increasing disease burden due to metabolic‐associated liver disease (MASLD) and alcohol‐associated liver disease (ALD), HCV‐related cirrhosis is still the greatest contributor to HCC [3, 17]. Less restrictive Medicaid policies for DAA prescriptions could therefore contribute to trends in HCV‐related HCC mortality. We utilized publicly available national mortality data to assess whether restrictive Medicaid DAA policies were associated with trends in HCC‐related deaths over time. We additionally performed a state‐by‐state analysis of Medicaid DAA policies and HCC‐related deaths before and after the introduction of DAAs to the market.

## 2 Methods

### 2.1 Data Source

CDC WONDER is a publicly available database that collects death certificate data for all US residents under the National Vital Statistics System. County‐level national mortality data from 1999 to 2023 is collected based on death certificates for US residents. Each death certificate identifies a single cause of death via ICD code. For estimation of crude and age‐adjusted death rates, population estimates are from the US Census Bureau. Age‐adjusted rates are based on 2000 standard US population [18, 19].

### 2.2 Medicaid Restrictions

We used the publicly available Hepatitis C: State of Medicaid Access state‐by‐state scoring system developed by the National Viral Hepatitis Roundtable and the Harvard Center for Health Law and Policy Innovation [7]. This system assigns states’ DAA accessibility grades with regard to prior authorization (PA) restrictions, categorized as follows: A + (PA removed for most patients; no other restrictions), A (PA removed for most patients or PA required for all patients; minimal restrictions), B (PA removed for most patients; some restrictions or PA required for all patients; minimal restrictions), C (PA required for all patients; some restrictions), D (PA required for all patients; many restrictions), and F (PA required for all patients; harsh restrictions) [7]. There were no F‐graded states in the February 2024 snapshot [7]. The state‐by‐state restrictions encompassed fibrosis levels (e.g., limiting therapy to patients with cirrhosis), substance use history, prescriber specialty or expertise, retreatment, prior authorizations, and access in managed care and additional restrictions [7]. We used the cross‐sectional report card from February 2024 and stratified states into three groups based on accessibility grade: A +/A (*n* = 28), B (*n* = 11), and C/D (*n* = 12) (Table 1).

**TABLE 1:** State of DAA Medicaid access: Overall state grades (February 2024).

A+, A^†^	B^†^	C, D^†^
(*n* = 28)	(*n* = 11)	(*n* = 12)
Alaska	Alabama	Arkansas
Arizona	Florida	Georgia
California	Kentucky	Iowa
Colorado	Minnesota	Maine
Connecticut	Mississippi	Maryland
Delaware	New Mexico	Montana
District of Columbia	Ohio	Nebraska
Hawaii	South Carolina	New Jersey
Idaho	Tennessee	Nevada
Illinois	Vermont	North Dakota
Indiana	Wyoming	Utah
Kansas		West Virginia
Louisiana		
Massachusetts		
Michigan		
Missouri		
New Hampshire		
New York		
North Carolina		
Oklahoma		
Oregon		
Pennsylvania		
Rhode Island		
South Dakota		
Texas		
Virginia		
Washington		
Wisconsin		

^†^ A+: PA removed for most patients; no restrictions, A: PA removed for most patients, or PA required for all patients; minimal restrictions, B: PA removed for most patients; some restrictions, or PA required for all patients; minimal restrictions, C: PA required for all patients; some restrictions, D: PA required for all patients; many restrictions, F: PA required for all patients; harsh restrictions [7].

Using the same Hepatitis C: State of Medicaid Access, we extracted state‐by‐state Medicaid accessibility restrictions for DAA prescriptions from 2014 and 2019–2024 [7]. Sub‐categories provided by this resource included fibrosis restrictions, substance use/sobriety restrictions, prescriber restrictions, PA restrictions, retreatment restrictions, managed care/additional restrictions [7]. We assessed the proportions of states that had each sub‐category of restrictions in 1‐year intervals over time.

### 2.3 HCC‐Related Death Rates

We extracted mortality data with HCC (C22.0) as a primary cause of death from 1999 to 2023 (all years included in the database). This included total HCC‐related deaths, total populations, crude death rates, and age‐adjusted death rates with respective 95% confidence intervals. We extracted data in state groupings (A + /A, B, C/D) and in individual states. Age‐adjusted death rate trends were determined based on age distributions from the 2000 U.S. census (20–29, 30–39, 40–49, 50–59, 60–69, 70–79, ≥ 80 years), utilizing methodologies recommended by the NCHS [18, 19].

### 2.4 Statistical Analysis

Age‐adjusted death rates were assessed for rate changes using the National Cancer Institute's Joinpoint program (version 5.3.0.0, http://surveillance.cancer.gov/joinpoint) [20]. A Joinpoint analysis yields annual percent change (APC) and average annual percent change (AAPC) for each group and identifies changes in death rate trends over time. Using piecewise linear regression with the least squares approach, the Joinpoint program produces slopes that describe the average APC in death rate. Changes in death rate over time are described as Joinpoints. APC and AAPC are calculated to carry statistical significance at an alpha of 0.05, with reported 95% confidence intervals. Crude death rates in the time periods of 1999–2014 and 2015–2023 were retrieved for each state and compared with absolute changes in crude death rates and relative percent change between crude death rates. These were organized by Medicaid accessibility group.

## 3 Results

### 3.1 Overall HCC Deaths Per Medicaid Accessibility Grade

From 1999 to 2023 group A + /A states had 151,806 HCC deaths, B had 44,331 HCC deaths, and C/D had 25,080 HCC deaths. According to 2022 US Census Bureau data, Group A + /A (28 states) contained approximately 66% of the US population (219,192,559), group B contained 20% (67,920,490), and group C/D contained 14% (46,174,508). In 2023, states with the highest crude death rates per 100,000 population were Rhode Island (5.9), Oregon (5.8), Louisiana (5.2), Alaska (5.2), and Hawaii (5.0). States with the lowest crude death rates in 2023 were Nebraska (2.4), Utah (2.4), New Jersey (2.6), South Dakota (2.6), and New York (2.9) (Table 2).

**TABLE 2:** State‐by‐state HCC‐related absolute difference and relative percent change in crude death rates between 1999 to 2014 and 2015 to 2023 organized by medicaid accessibility grade.

Medicaid accessibility grade	State	Total deaths 1999–2023	Total deaths 1999–2014	Crude death rate 1999–2014	Total deaths 2015–2023	Crude death rate 2015–2023	Absolute difference in crude death rate	Relative percent change (%)
A+/A	Alaska	546	285	2.62	261	4.08	1.46	55.73
	Arizona	4523	2234	2.34	2289	3.54	1.20	51.07
	California	29,998	17,000	2.93	12,998	3.69	0.76	25.94
	Colorado	3168	1487	1.94	1681	3.31	1.37	70.62
	Connecticut	2472	1447	2.57	1025	3.17	0.60	23.35
	Delaware	750	374	2.72	376	4.12	1.40	51.29
	District of Columbia	688	380	4.01	308	4.74	0.73	18.20
	Hawaii	1575	897	4.28	678	5.23	0.94	22.08
	Idaho	1010	455	1.94	555	3.46	1.52	78.09
	Illinois	7377	4078	2.01	3299	2.95	0.94	46.77
	Indiana	4127	2150	2.12	1977	3.26	1.14	53.54
	Kansas	1681	822	1.84	859	3.24	1.40	75.82
	Louisiana	3136	1475	2.04	1661	4.16	2.12	103.68
	Massachusetts	5342	3095	2.98	2247	3.66	0.68	22.65
	Michigan	6435	3413	2.14	3022	3.41	1.27	59.35
	Missouri	4099	2186	2.34	1913	3.51	1.17	50.00
	New Hampshire	903	490	2.37	413	3.33	0.96	40.30
	New York	12,703	7620	2.47	5083	2.88	0.41	16.40
	North Carolina	7411	3558	2.48	3853	4.09	1.61	64.92
	Oklahoma	2638	1129	1.94	1509	4.33	2.39	122.94
	Oregon	3724	1710	2.9	2014	5.37	2.47	85.17
	Pennsylvania	9792	5229	2.61	4563	3.96	1.35	51.72
	Rhode Island	880	442	2.61	438	4.59	1.98	75.86
	South Dakota	437	189	1.49	248	3.22	1.73	115.77
	Texas	21,149	10,493	2.77	10,656	4.16	1.39	50.18
	Virginia	5404	2789	2.27	2615	3.46	1.19	52.42
	Washington	6119	3125	3.04	2994	4.42	1.38	45.23
	Wisconsin	3719	1930	2.16	1789	3.46	1.30	60.19
B	Alabama	2689	1421	1.91	1268	2.99	1.08	56.28
	Florida	14,593	7641	2.66	6952	3.60	0.94	35.34
	Kentucky	2889	1391	2.06	1498	3.92	1.86	90.05
	Minnesota	3388	1742	2.1	1646	3.21	1.11	52.86
	Mississippi	1224	569	1.22	655	2.58	1.36	111.07
	New Mexico	1914	951	3.03	963	5.08	2.05	67.49
	Ohio	8007	4141	2.25	3866	3.71	1.46	64.67
	South Carolina	3560	1685	2.4	1875	4.12	1.72	71.46
	Tennessee	5202	2571	2.63	2631	4.34	1.71	64.83
	Vermont	488	242	2.44	246	4.31	1.87	76.43
	Wyoming	377	169	1.98	208	4.00	2.02	102.02
C/D	Arkansas	1469	569	1.26	900	3.47	2.21	175.40
	Georgia	5322	2498	1.7	2824	3.03	1.33	78.24
	Iowa	1872	948	1.98	924	3.33	1.35	68.18
	Maine	981	535	2.55	446	3.69	1.14	44.71
	Maryland	4210	2203	2.45	2007	3.64	1.19	48.57
	Montana	701	317	2.07	384	3.93	1.86	89.86
	Nebraska	910	499	1.75	411	2.41	0.66	37.43
	Nevada	1692	795	2.01	897	3.29	1.28	63.43
	New Jersey	5347	3210	2.31	2137	2.64	0.33	14.29
	North Dakota	334	156	1.47	178	2.57	1.10	74.49
	Utah	1095	510	1.24	585	2.10	0.86	69.35
	West Virginia	1147	565	1.93	582	3.69	1.76	90.93

State‐by‐state HCC crude death rates were obtained between 1999 to 2014 and 2015 to 2023 with comparison calculations of absolute difference in crude death rates and relative percent changes. All states had an increase in the absolute difference in crude death rates ranging from 0.33 (New Jersey) to 2.47 (Oregon). The lowest relative percent changes in HCC‐related crude death rate were seen in New Jersey (14.3%), New York (16.4%), Massachusetts (22.7%), and Connecticut (23.4%) (Table 2).

### 3.2 Medicaid Accessibility Grades From 2014 to 2024

From 2014 to 2024, data were collected in available sub‐categories: fibrosis restriction, sobriety use restriction, and prescriber restrictions. From 2022 to 2024, additional sub‐categories included PA restrictions, retreatment restrictions, and managed care/additional restrictions. By 2024, 100% of states had eliminated fibrosis restrictions, 86% had eliminated sobriety/substance use restrictions, 94% had eliminated prescriber restrictions, 74.5% had eliminated retreatment restrictions, and 78% had eliminated managed care/or additional restrictions (Table 3). With yearly sampling, all states experienced improved accessibility for DAA medications. Therefore, selecting a snapshot of the 2024 prescription landscape as a baseline comparator would be the most forgiving for our analysis in categorizing states.

**TABLE 3:** DAA Medicaid accessibility with restriction sub‐categories over time.

	2014	2019	2020	2021	2022	2023	2024
N (States)	51 (14.3%)	51 (14.3%)	51 (14.3%)	51 (14.3%)	51 (14.3%)	51 (14.3%)	51 (14.3%)
Fibrosis Restriction							
No	0 (0.0%)	38 (74.5%)	45 (88.2%)	47 (92.2%)	49 (96.1%)	50 (98.0%)	51 (100.0%)
Yes	34 (100.0%)	13 (25.5%)	6 (11.8%)	4 (7.8%)	2 (3.9%)	1 (2.0%)	0 (0.0%)
Sobriety/Substance Use restriction							
No	0 (0.0%)	14 (27.5%)	14 (27.5%)	23 (45.1%)	32 (62.7%)	41 (80.4%)	44 (86.3%)
Yes	36 (100.0%)	37 (72.5%)	37 (72.5%)	28 (54.9%)	19 (37.3%)	10 (19.6%)	7 (13.7%)
Prescriber Restriction							
No	0 (0.0%)	22 (43.1%)	24 (47.1%)	33 (64.7%)	40 (78.4%)	47 (92.2%)	48 (94.1%)
Yes	29 (100.0%)	29 (56.9%)	27 (52.9%)	18 (35.3%)	11 (21.6%)	4 (7.8%)	3 (5.9%)
PA Restriction							
No					14 (27.5%)	25 (49.0%)	29 (56.9%)
Yes					37 (72.5%)	26 (51.0%)	22 (43.1%)
Retreatment Restriction							
No					28 (54.9%)	34 (66.7%)	38 (74.5%)
Yes					23 (45.1%)	17 (33.3%)	13 (25.5%)
Managed Care/Additional Restrictions							
No					19 (50.0%)	27 (65.9%)	32 (78.0%)
Yes					19 (50.0%)	14 (34.1%)	9 (22.0%)

### 3.3 Age‐Adjusted Death Rates in 1999 and 2023

**FIGURE 1:**
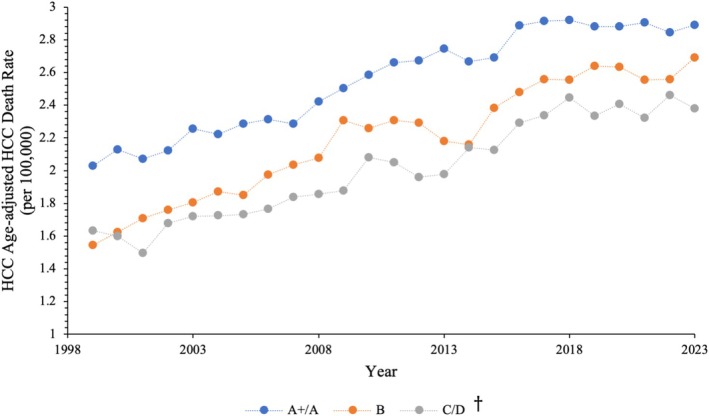
Trends in age‐adjusted HCC death rate in patients stratified by DAA Medicaid accessibility grade (1999–2023). ^†^DAA Medicaid accessibility grade (February 2024).

In 1999, the age‐adjusted HCC‐related death rate for the three groups per 100,000 population was as follows: 2.03 (95% CI: 1.96, 2.10) in group A+/A, 1.54 (95% CI: 1.44, 1.65) in group B, and 1.63 (95% CI: 1.50, 1.77) in group C/D. Between 1999 and 2023, the highest burden of age‐adjusted HCC mortality was observed in A+/A states followed by B and C/D states. Trends followed rising trajectories during this time period (Figure 1).

### 3.4 Changes in Age‐Adjusted Death Rates: APC And AAPC

**FIGURE 2:**
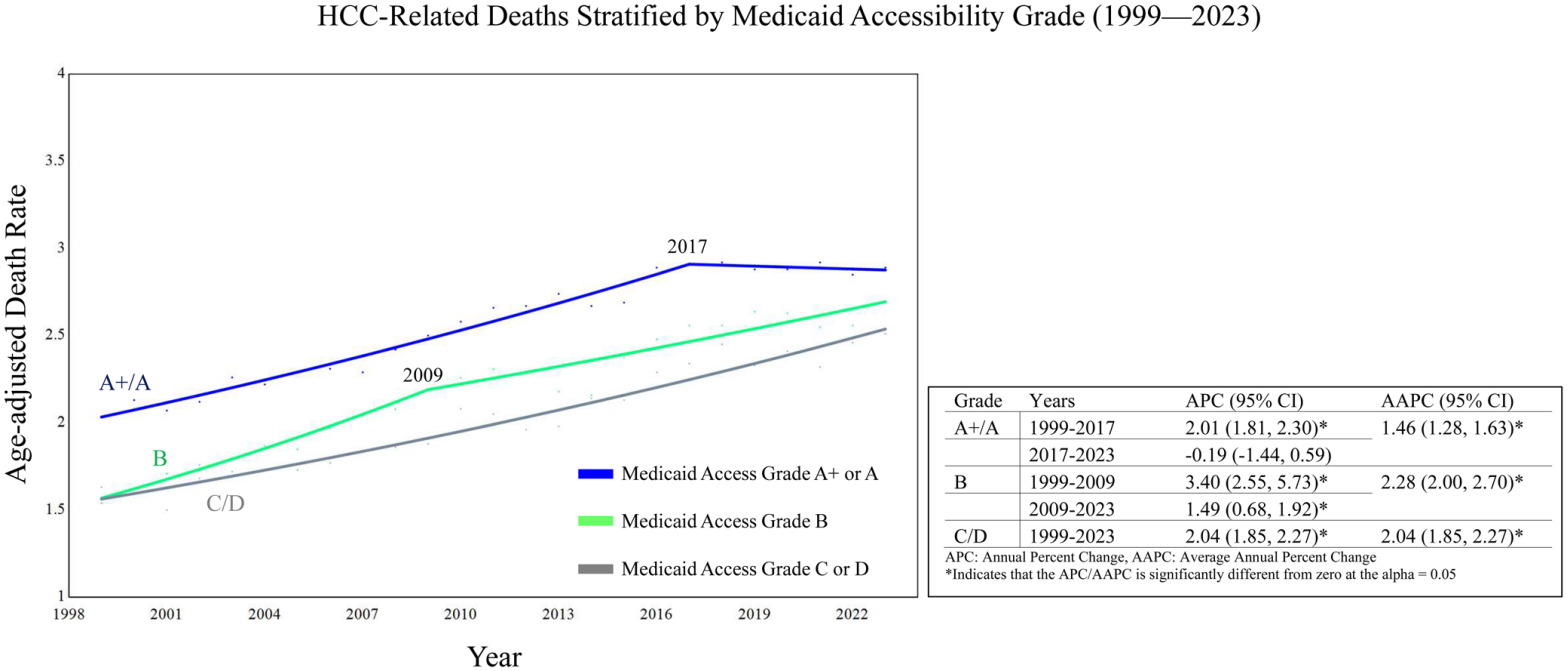
Joinpoint analysis of HCC age‐adjusted death rate stratified by DAA Medicaid accessibility grade (1999–2023).

**FIGURE 3:**
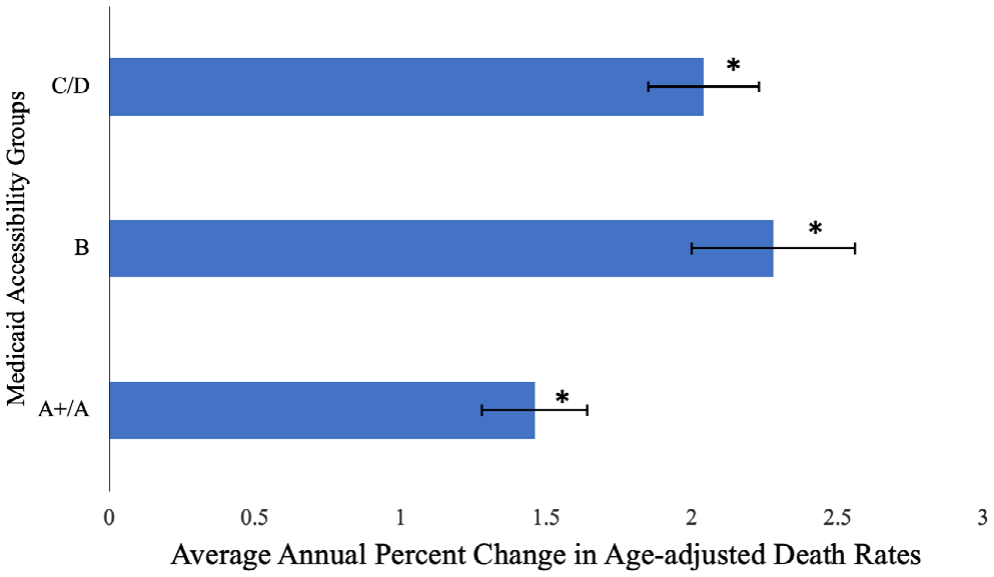
Average annual percent change (AAPC) in HCC age‐adjusted death rates (1999–2023). *Statistically significant difference from zero, alpha = 0.05.

Prior to 2017, the APC in HCC‐related death rates was positive in all groups. After 2017, APC became negative only in group A+/A, APC = −0.19 (95% CI: −1.44, 0.59) although this APC was not significantly different from zero. In contrast to group A+/A, the APC for groups B and C/D remained consistently positive throughout the interval, but group B states had a significant decrease in their positive APC rate in 2009: 1999–2009 APC = 3.40 (95% CI: 2.55, 5.73) and 2009–2023 APC = 1.49 (95% CI: 0.68, 1.92). Group C/D states’ APC was 2.07 (95% CI: 1.85, 2.32) (Figure 2). The AAPC from 1999 to 2023 in HCC age‐adjusted death rates was 1.46 (95% CI: 1.28, 1.63) in A +/A states, which was significantly lower than AAPCs for group B states: 2.28 (95% CI: 2.00, 2.70) and group C/D states: 2.04 (95% CI: 1.85, 2.27) (Figure 3).

## 4 Discussion

Primary liver cancer, predominantly HCC, is projected to become the third leading cause of cancer‐related death by 2035 and HCV remains the primary cause of HCC [3, 21, 22]. Insurance with Medicaid is associated with more advanced HCC stage at diagnosis, lower treatment rates, and increased mortality [23, 24]. Primary prevention of HCC by treating HCV with DAAs has the potential to reduce HCC incidence, downstream deaths, and is recommended by the *AASLD* guidelines as a primary prevention strategy [3]. Additionally, screening and treating HCV is cost‐effective in both adults and adolescent patients in the long term [2, 25]. Our analysis suggests that fewer Medicaid restrictions for DAA access are associated with a greater reduction in HCC‐related deaths.

The costs of DAAs and resulting insurance barriers to access have created disparities in HCV treatment and cure [1]. HCV patients who undergo DAA treatment and achieve sustained virologic response (SVR) have a significantly reduced risk of HCC [8]. In a study of Arizona Medicaid beneficiaries between 2013 and 2019, only 13.3% of HCV patients received DAAs, but among those who were treated, there was an association with a lower risk of HCC (compensated cirrhosis), lower risk of liver‐related mortality, and lower all‐cause mortality [26]. In the VHA system, where HCV screening and treatment campaigns have been highly successful, data coinciding with the introduction of DAAs demonstrated a reduction in HCV‐related HCC incidence from 2015 to 2018 and improved overall survival [14, 15]. A national cross‐sectional study of Medicaid claims (*n* = 381,373) examined the association between prior authorization (PA) restrictions and Medicaid non‐expansion status on DAA access [5]. Claims data demonstrated that Medicaid non‐expansion status, fibrosis restrictions, and sobriety restrictions were associated with a lower likelihood of DAA treatment for Medicaid beneficiaries [5].

Our study builds upon these findings by assessing whether state‐by‐state Medicaid restrictions for DAA access are associated with a change in HCC mortality over time. Given that Medicaid DAA restrictions liberalized over time (Table 3), a later snapshot would provide an inclusive timepoint for state categorization. These data suggest that after 2017, fewer PA restrictions on DAAs were associated with a declining incidence rate of HCC deaths. In states graded A + /A, there was a decline in the APC in age‐adjusted death rates and a significantly lower AAPC in death rates from 1999 to 2023 compared to states graded B or C/D. This provides further evidence that Medicaid expansion and improved accessibility to DAAs are associated with a decline in HCV‐related HCC incidence and mortality.

13.7% and 25.5% of states continue to enforce sobriety/substance use and retreatment restrictions, respectively, for Medicaid beneficiaries needing DAAs (Table 3). National guidance and a significant body of evidence suggest drug use or concern for reinfection is not a contraindication for HCV treatment [6]. This is based on multiple clinical trials and observational studies that demonstrate SVR rates approaching 95% in PWID at the start of or during HCV therapy [6, 16, 27, 28, 29, 30]. In addition to offering DAA treatment, the optimal approach to this patient population should include counseling to reduce risk of transmission, linkage to harm reduction medications and services (naloxone, needle/syringe service programs, medications for opioid use disorder, substance use disorder treatment programs) [6, 31].

There are several limitations to this study. Given its cross‐sectional, retrospective nature, many possible confounders can exist in HCC‐related mortality data that could underlie Medicaid DAA access, such as improved advanced therapies for HCC, improved detection of HCV or HCC, differences in demographics between grouped states, access to cirrhosis sub‐specialized care, and sample size differences between grouped states. There is potential for mortality misclassification in death certificate data [32]. In addition, there is a potential regression to the mean since A +/A grade states started with a higher HCC age‐adjusted death rate in 1999. Although the leading contributor, HCC‐related deaths, is driven by more than just HCV, there is also a relative increase in ALD‐related and MASLD‐related HCC incidence in the US [17]. The CDC WONDER dataset provides overall HCC‐related deaths based on the primary and contributing causes of death noted on death certificates, but this data source does not allow us to assess if changes in HCC deaths are attributable to specific causes such as a decreased incidence of HCC, lower risks of HCC recurrence, or decreased liver‐related decompensation risks among patients with HCC. Furthermore, this dataset does not offer patient‐level details to allow for comparisons by important covariates such as HCV treatment status, ongoing alcohol use, PWID, or liver fibrosis [18, 19]. Overall HCV infection is skewing toward younger patient populations and these patients may have milder disease and a lower risk for HCC‐related mortality [33]. We hope that by presenting age‐adjusted HCC‐related data, this would mitigate effects of such factors. These national mortality data demonstrate an association between declining DAA Medicaid restrictions and declining HCC mortality. DAA Medicaid restrictions declined from 2014 to 2024, but individual patient‐level data for HCV treatment rates directly linked to policy changes and subsequent HCC incidence is not available in this database. Further studies could assess patient‐level data comparing DAA‐SVR treated groups with analyzing at‐risk populations (alcohol consumption, PWID, and liver fibrosis scores) and HCC incidence.

In conclusion, states with less restrictive access to DAA medications were associated with an improvement in HCC‐related mortality following 2017. The proposed 5‐year program to eliminate HCV, with improved access to DAAs as a main solution, has yet to achieve legislative funding [4]. While these data cannot establish causation between DAA access and HCC mortality, we hope this evidence can add to the impetus of continued policy reform in Medicaid expansion and PA requirements in DAA access.

## Author Contributions

**Gabriel V. Lupu:** conceptualization (equal), data curation (equal), formal analysis (equal), investigation (equal), methodology (equal), visualization (equal), writing – original draft (equal), writing – review and editing (equal). **Bhagyashree Behera:** data curation (supporting), writing – review and editing (supporting). **A. Sidney Barritt IV:** conceptualization (equal), supervision (equal), writing – review and editing (equal). **Sasha Deutsch‐Link:** writing – review and editing (supporting). **Jane Giang:** writing – review and editing (supporting). **Ellen W. Green:** writing – review and editing (supporting). **Oren K. Fix:** writing – review and editing (supporting). **Neil D. Shah:** writing – review and editing (supporting). **Hersh Shroff:** writing – review and editing (supporting). **Andrew M. Moon:** conceptualization (equal), formal analysis (supporting), investigation (supporting), methodology (supporting), supervision (equal), writing – review and editing (equal).

## Funding

This work was supported by the National Institutes of Health, T32 DK 007634.

## Ethics Statement

This project was exempt from IRB review and informed consent since it is limited to secondary analysis of a publicly available, de‐identified data source.

## Conflicts of Interest

Andrew M. Moon: Consulting TARGET RWE, Intercept Pharmaceuticals, IDEOlogy Health. Research funding (to institution) from DCN diagnostics.

A. Sidney Barritt 4th: Consulting Madrigal, Mirium, Merck, BI, LifeEdit, Target RWE.

Sasha Deutsch‐Link: Consulting Nanbar Health.

Neil D. Shah: Consulting for GSK and Ipsen.

For other authors, there are no additional disclosures or potential conflicts of interest.

## Data Availability

The data that support the findings of this study are openly available in “CDC WONDER” (https://wonder.cdc.gov/) and “Hepatitis C State of Medicaid Access” (https://stateofhepc.org/).
